# Abnormal eruption of teeth in relation to *FGFR1* heterozygote mutation: a rare case of osteoglophonic dysplasia with 4-year follow-up

**DOI:** 10.1186/s12903-022-02069-6

**Published:** 2022-02-11

**Authors:** Yuchun Zou, Hanyu Lin, Weijia Chen, Lin Chang, Senxin Cai, You-Guang Lu, Linyu Xu

**Affiliations:** 1 Fujian Key Laboratory of Oral Diseases and Fujian Provincial Engineering Research, Center of Oral Biomaterial and Stomatological Key Lab of Fujian College and University, School and Hospital of Stomatology, Fujian Medical University, Fuzhou, China; 2 Institute of Stomatology and Research Center of Dental Esthetics and Biomechanics and Department of Orthodontics, School and Hospital of Stomatology, Fujian Medical University, Fuzhou, China; 3 Department of Preventive Dentistry, Fujian Key Laboratory of Oral Diseases, School of Stomatology, Fujian Medical University, Fuzhou, 350001 China; 4 Department of Orthodontics, Fujian Key Laboratory of Oral Diseases, School of Stomatology, Fujian Medical University, Fuzhou, 350001 China

**Keywords:** Osteoglophonic dysplasia, Skeletal and dental disorder, *FGFR1*, Impacted teeth, Genetic mutation, Case report

## Abstract

**Background:**

We report a case and its 4-year follow-up of Osteoglophonic dysplasia (OD), a rare disease that disturbs both skeletal and dental development, which is usually caused by heterozygous *FGFR1* mutations.

**Case presentation:**

This article presents a case where a 6-year-old male patient suffered dysregulation of tooth eruption and was diagnosed with osteogenic dysplasia from a fibroblast growth factor receptor 1 (*FGFR1*) heterozygote mutation. However, the number of teeth is within the normal range, and their roots are well developed. Several interventions were implemented with varying degrees of results. The details of the 4-year follow-up showed that the signs of OD were more pronounced, including dwarfism, frontal bossing, delayed skeletal maturation, anteverted nares, micrognathia, and prominent ears, but the patient’s impacted teeth and edentulous jaws remained unchanged.

**Conclusions:**

*FGFR1* heterozygote mutation and OD present significant difficulty for teeth eruption and subsequent intervention. Further measures ought to be taken in recognizing various symptoms presented by the patient. This case supports the significance of careful inquiry, comprehensive physical examination and correct diagnosis as indispensable steps for clinical practice in patients with unerupted teeth. Additionally, the detailed case and its 4-year follow-up length may provide new insights into osteogenic dysplasia and patients with impacted teeth while encouraging further exploration in treatment methods.

**Supplementary Information:**

The online version contains supplementary material available at 10.1186/s12903-022-02069-6.

## Background

The fibroblast growth factor *(FGF*) genes and their receptors (*FGFRs*) serve considerable roles in both dental and skeletal development [1, 2]. *FGFR1*-*4* expression in osteoblasts and chondrocytes affects the proliferation and differentiation of cells and participates in the regulation process of bone tissue, especially axial and craniofacial skeleton; in addition, *FGFR1* is also expressed in osteoclasts and works with *FGFR2* to regulate osteoclastogenesis [3].

Osteoglophonic dysplasia (OD) (OMIM number: # 166250), caused by *FGFR1* mutation, is a rare disease that is characterized by stunting of stature, craniosynostosis, facial dysmorphism, brachydactyly stubby fingers and toes [2–4]. To date, however, only sporadic cases of OD have been reported since it was first described in 1951, and few studies have focused on intraoral changes [5].

In literature, researchers have studied patients with OD, from age 1 day to 25 years [6–8]. Patients suffering from OD share the salient features of craniofacial and dental problems persist throughout their life. Since OD was first reported in 1951, its radiographic features, biological characteristics, and clinical manifestations have been gradually recognized. An abnormal form of the vertebral bodies, craniosynostosis (frontal bossing) with tower-shaped cranium (clover leaf skull) or oxycephaly, hypertelorism (wide-set eyes), severe short stature (dwarfism) and multiple unerupted teeth are commonly found in almost all patients with OD [6, 9–12]. Symptoms, including abnormal collarbone, delayed skeletal maturation, anteverted nares, micrognathia, prominent ears and rhizomelia, may occur in 30–79% of people with OD. Abnormal bone ossification, brachydactyly, choanal atresia, cryptorchidism, inguinal hernia and scoliosis may also appear in some patient cases.

Additionally, previous case reports also reveal OD characterized radiographically by extraordinarily thinning cortical bone, even cortical defects, metaphyseal dysplasia, lower ossein density and multiple cystoid radiolucent foci, which means the bones are brittle and prone to fracture with severe osteoporosis [7, 10, 13, 14]. Concerning laboratory examinations of hematological and biochemical studies, the levels of potassium, calcium, sodium and lipoproteins are usually within normal ranges, except low phosphate and elevated alkaline phosphatase are common anomalies in some cases [9, 15, 16].

Studies disclose that what causes OD syndrome are mutations in the *FGFR1* gene which controls the growth of bone and tooth formation and eruption [2–4]. Cells, including mesenchymal progenitors, hypertrophic chondrocytes and differentiated osteoblasts, express *FGFR1* in the formation of perichondrium, collar and trabecular bone, mesenchymal condensation mediation, mineralization of the bone and endochondral and intramembranous bone formation. Disorders in the expression pattern of *FGFR1* can induce craniosynostoses and chondrodysplasias [17]. *FGFR1* conditional knockout mice present impaired chondrocyte hypertrophy at the early stage of bone development. Conversely, an expanded hypertrophic chondrocyte zone is found in the later stage [18]. Therefore, patients with OD usually have manifestations of depressed nose, helical ears, flattening condyle and metaphysis dysplasia, which are associated with changes in the *FGFR1* gene during chondrogenesis. Indeed, *FGFR1* is also crucial for long bone development, and the limbs of patients with OD are susceptible to cystic lesions and non-ossifying fibroids, finally resulting in dwarfism and sometimes inducing pseudoarthrosis [9, 10]. In terms of their role in tooth development, mutations at the site of *FGFR1*, on the one hand, tend to prevent the eruption of both deciduous and permanent teeth, which may be due to the reversing and crookedness of teeth, the impaction of hyperplastic gingiva, or even jaw cysts [2, 11]. On the other hand, they also affect the number of tooth buds, resulting in the oligodontia recorded in the literature [7, 11].

In this case report, we present a 4-year follow-up of a boy with dysregulation of eruption of teeth who was diagnosed with OD related to *FGFR1* heterozygote mutations.

## Case presentation

On June 11, 2016, at the age of 6 years and 7 months, the patient visited the Department of Orthodontics in our hospital seeking treatment for abnormal eruption of his teeth.

### Medical history

#### Family history

The patient had a negative family history with no relevant diagnosis and no serious intraoral findings. Both his father and mother had good oral condition with normal numbers and well-developed tooth shapes as well as good occlusion (Additional file 1: Fig. S1, Additional file 2: Fig. S2).

#### Medical history

With custodian recollection, the patient had been to multiple visits for the aberrant manifestations of his teeth and skeletal development before visiting our department. The patient was a full-term male newborn with a weight of 4030 g and height of 51 cm. The patient’s first medical attention was due to his protruding forehead at age 1 year old. The patient then was diagnosed of rickets and presented with the finding of increased levels of alkaline phosphatase and delayed closure of cranial sutures. Previous hyper-osteogenic bone mineral density examination showed mild bone strength deficiency in the distal radius with an ultrasonic velocity of 3521 m/s at the age of 4 years and 10 months old. The patient received pharmacological treatment for rickets, and at the age of 6, he was cured from rickets.

### Symptoms

#### Physical presentation

**Fig. 1:**
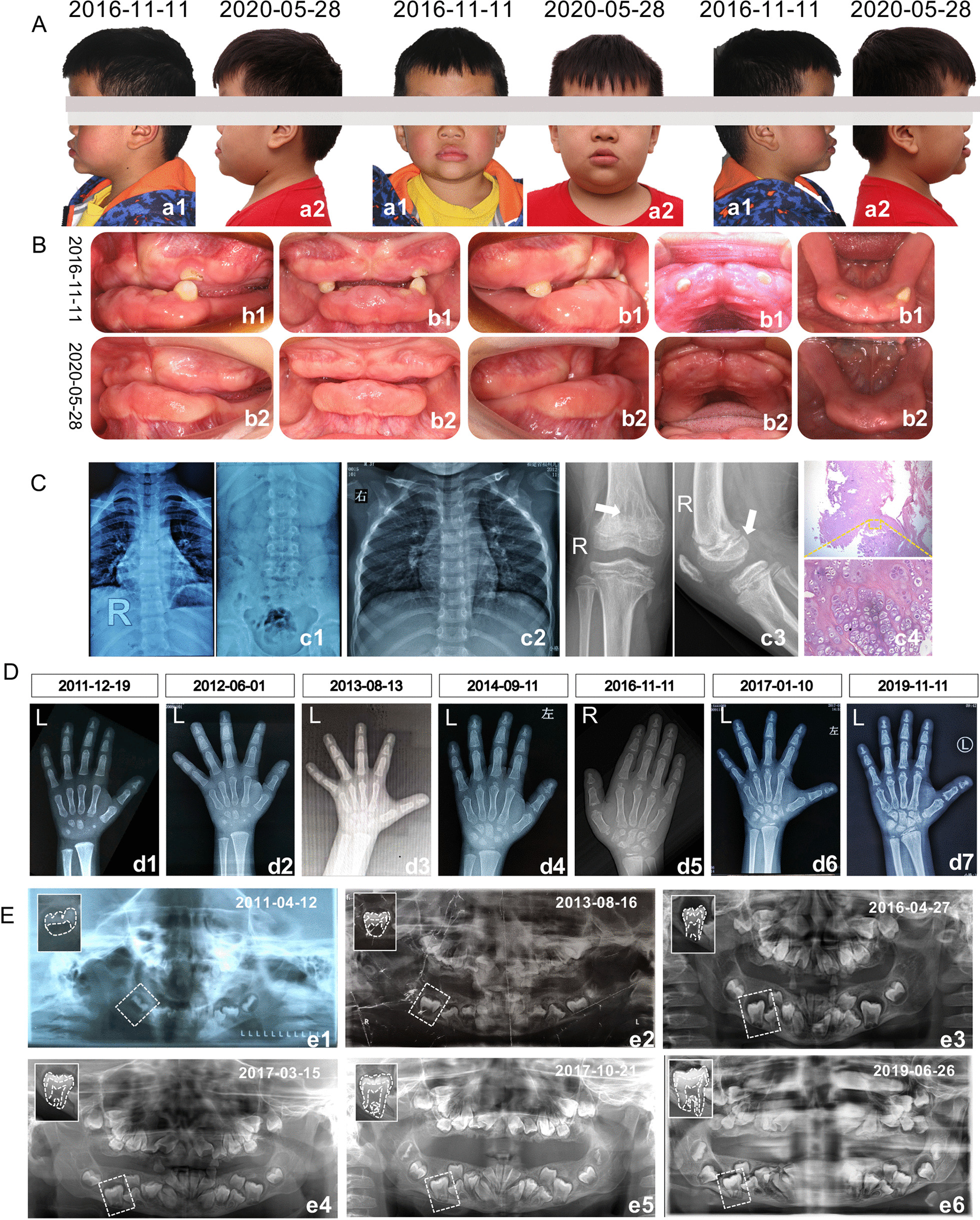
Intraoral and facial photographs and radiographical films. Facial (**A**) and intraoral (**B**) photos of the first visit (a1, b1) and 4-year follow-up (a2, b2). Films of the patient’s thoracolumbar (c1), chest (c2) and knee (c3) as well as histomorphometry of his leg chondroma stained with hematoxylin and eosin (c4). Series of his wrist radiographs (**D**) and panorama (**E**) ranging from 1 year old until now

The patient presented with a bossing forehead and protruding mandible as well as protruding lips. His midface hypoplasia was marked with a flattened nasal bridge, crumpled helices with protruding ears, and his skull presented oxycephaly (Fig. 1A, a1). His response to verbal command was normal; when interviewed, the patient performed well and responded quickly. In addition, no sounds could be heard while opening his mouth with no other abnormality in his temporomandibular joint.

#### Intraoral examination

On intraoral examination, few small and conical teeth was found with hypertrophic gingiva. There were only 5 deciduous teeth in the mouth, two in the upper jaw and 3 in the lower jaw, and most of them were surrounded by hypertrophied gingiva with a small portion of the crowns exposed clinically (Fig. 1A, b1). Two primary incisors had prematurely fallen out one year back, which indicated that until now, only seven of his primary teeth had erupted since the eruption of his first deciduous tooth at the age of 6 months.

#### Radiological presentation

The panoramic revealed that the patient’s teeth were inverted with delayed permanent tooth bud formation, along with flattening condyles (Fig. 1E).

In addition to his abnormal dental condition, the patient’s bone development was also dysplastic. Fusion between the trapezium and trapezoid, as well as the capitate and scaphoid, was seen in his hand on wrist radiograph, corresponding to a bone age of 11–12 years. In addition, the triquetrum bone was vaguely visible, but the sesamoid of the adductor pollicis was absent (Fig. 1D, d4). His 3-year-old hand and wrist radiograph displayed 7 carpal bones and both proximal phalangeal epiphyses and distal radial epiphysis without fusion (Fig. 1D, d3). Taken together, the results indicated that his bone age might be older than his physical age. It was particularly noteworthy that the shaft of his distal phalanx was extremely concave, and the shape of his epiphyses was especially thin and small, which is different from normal.

### Diagnosis

The diagnosis of OD was made on the basis of clinical manifestations, DNA sequencing, medical history and physical examination as well as reports from reported studies.

**Fig. 2:**
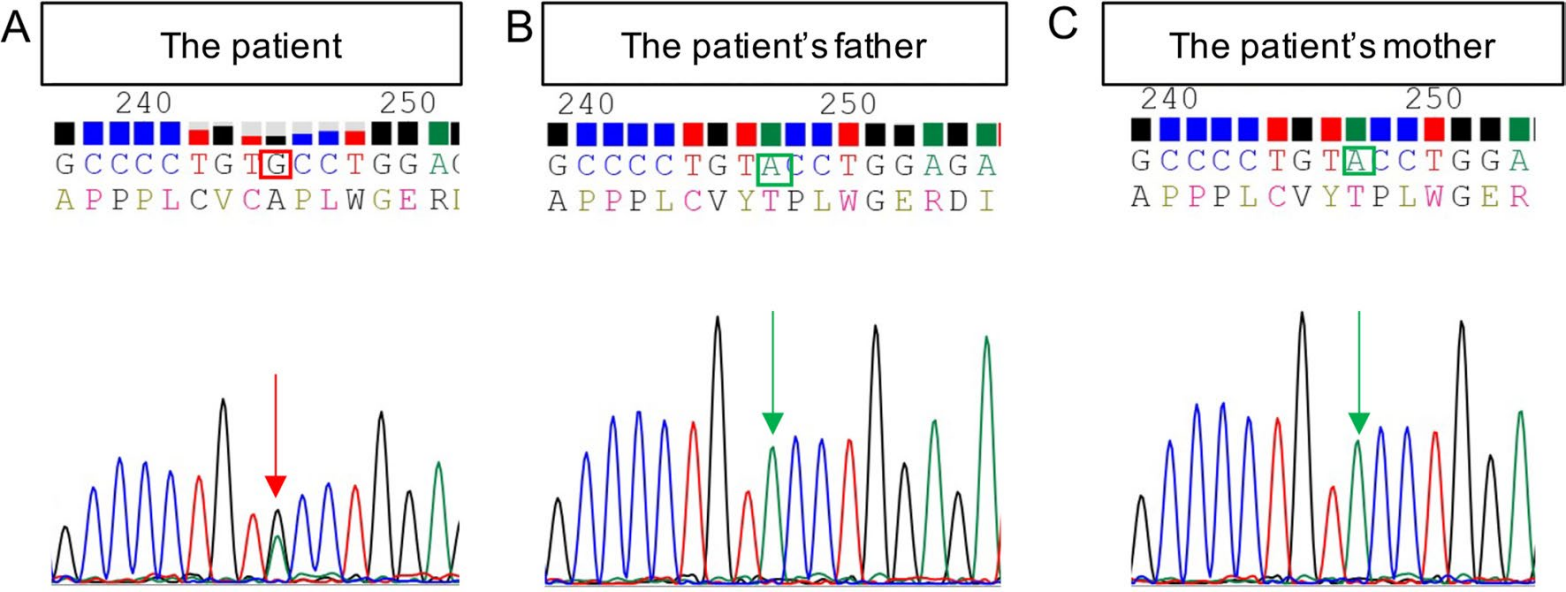
The exon gene sequencing results of the patient and his  parents, demonstrating *FGFR1* (NM 023110.2) heterozygous mutation c.1121A>G in exon 9 in the patient (**A**), while his mother (**B**) and father (**C**) are normal

In 2017, the patient undergone sequencing of the exon gene which demonstrated the *FGFR1* (NM 023110.2) heterozygous mutation c.1121A>G in exon 9 and induced a substitution of tyrosine 374 to cysteine (Tyr374Cys), while there was nothing abnormal with his mother’s genes and his father (Fig. 2). As a result, it was spontaneous.

For differential diagnosis, it is worth mentioning that patient suffers from the developmental disorders of bones and teeth with hypoplasia of the middle of the face, bulging forehead, which are common symtoms not only to OD, but also the other diseases such as Cleidocranial Dysplasia (CCD) (OMIM number: # 119600) and Hypohidrotic Ectodermal Dysplasia (HED) (OMIM number: # 305100) and so on [19–22]. Yet, CCD is a rare autosomal dominant skeletal disorder characterized by abnormal shape and number of teeth (usually are supernumerary teeth), and 65–75% cases showed mutations in runt-related transcription factor 2 [19, 20]. The case above does not fit any of the diagnostic symptoms. On the other hand, while abnormal development of ectoderm tissue is an important feature of a group of heterogeneous genetic diseases such as ED syndrome, oligo-anodontia, hypertrichosis, hypo-anhidrosis are characteristic triad of X linked HED, which means patients with HED usually have symptoms of dry skin, hair, and nail disorders besides missing teeth [21, 22]. In our case, such symptoms were not present. With differential diagnosis, the results of gene sequencing enabled a definitive diagnosis in our case.

### Intervention

On the basis of the patient’s oral and radiographical examinations, we suspected abnormal growth in both teeth and bone development.

The first intervention to assist the unerupted maxillary deciduous incisor to erupt was by cutting the gums to expose the crown when the patient was 3 years and 4 months old. The intervention ultimately failed, as the incision was quickly wrapped with the gum after 3 weeks.

Following the diagnosis of OD, at this time, due to the patient age and developmental condition as well as relatively few literature on the course of the patient disease, we and the patient custodian agreed upon awaiting further follow-ups while implementing prosthetic rehabilitation (removable denture) to ease the pain, to assist the patient in daily mastication, and to prevent mandibular prognatism due to edentulous jaw, as well as to reduce possible social impact due to appearance at this stage (Additional file 3: Fig. S3).

### Follow-up

**Fig. 3:**
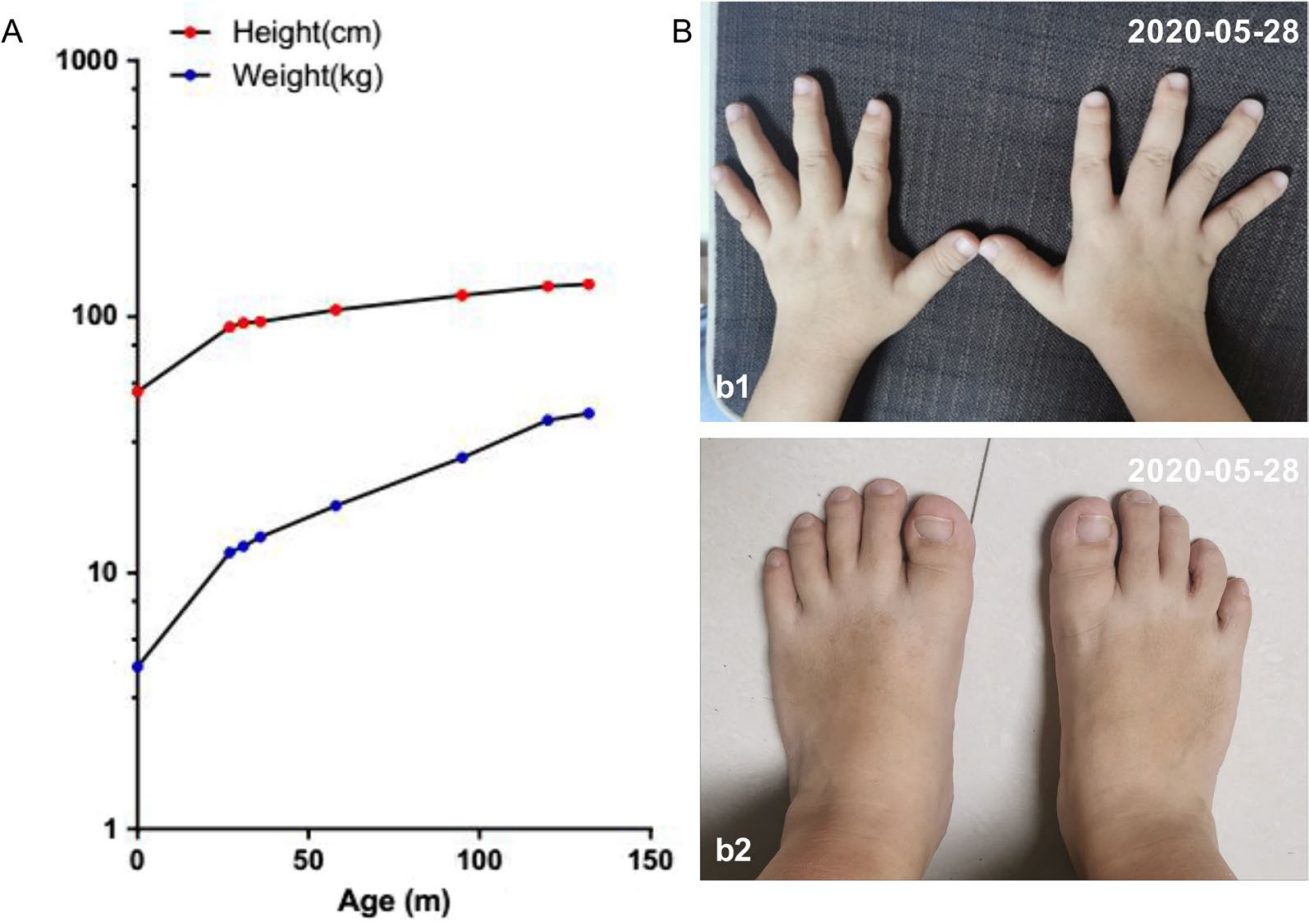
Growth condition of the patient. **A** Growth charts of the patient. **B** Photos of his fingers (b1) and toes (b2)

The patient condition did not improve in the following four years of follow-up (Fig. 1A, a2, b2). Five deciduous teeth had all been lost, but no permanent teeth had emerged based on the follow-up records on June 11, 2019; instead, only hyperplastic inflamed gingiva could be seen on the thick and bulbous alveolar ridges, and a high palatal vault and a large tongue were found in his mouth. Notably, his dwarfism became more obvious with elbow and knee shrinkage (Fig. 3, Table 1).

**Table 1:** Records of the patient’s heights (cm) and weights (kg)

Age (m)	0	27	31	36	58	95	120	132
Weight (kg)	4.3	12	12.7	13.8	18.3	28.2	39.5	42
Length/height (cm)	51	91.2	94.6	95.7	106.5	121.3	131.5	134

**Fig. 4:**
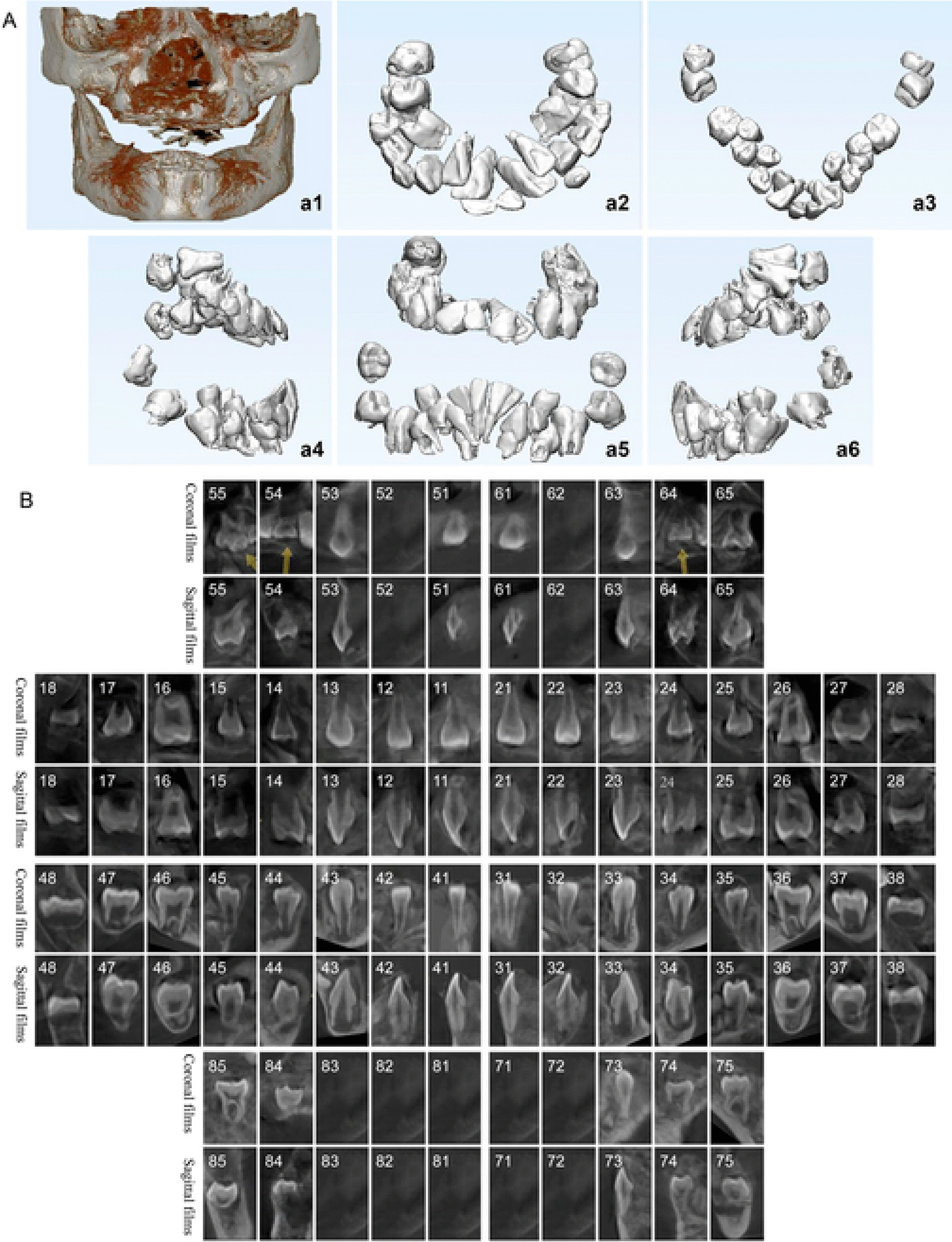
Cone-beam computed tomography (CBCT) images of his teeth with both three-dimensional reconstructed images (**A**) and two-dimensional images of the coronal and sagittal planes of each tooth (**B**)

We analyzed his cone-beam computerized tomography (CBCT) on June 26, 2019, and found that the number of his teeth was normal, with a total of 32 permanent tooth buds and 12 deciduous teeth impacted in the alveolar bone, and the other 7 erupted deciduous teeth had shed (Fig. 4). Dental age was 8.5 years old, as determined by Demirjian’s method, which was younger than his chronological age of 9 years 7 months (Table 2). The enamel seemed to be poorly developed with relatively decreased thickness (Table 3).

**Table 2:** Dental age determined by Demirjian’s method

Tooth type	I1	I2	C	PM1	PM2	M1	M2
Operator 1							
Stages	H	F	G	E	F	F	E
Scores	11.8	7.8	11	11	12.8	12.3	12.5
Operator 2							
Stages	H	H	F	F	F	E	D
Scores	11.8	13.7	10	12.3	12.8	9.6	10.1
Mean total scores from two operators	(79.2 + 80.3)/2 = 79.75						
Dental age	8.5 years old						

I1, central incisor; I2, lateral incisor; C, canine; PM1, first premolar; PM2, second premolar; M1, first molar; M2, second molar

**Table 3:** Enamel thickness (mm) of patient’s teeth measured from his CBCT on June 26, 2019

Tooth	15	14	13	12	11	21	22	23	24	25
Buccal Cusp	2.8	2.55	1.85	2.5	2.2	2.2	2.65	1.75	1.5	1.7
Palatal Cusp	2.35	1.75	–	–	–	–	–	–	0.80	2.7

Tooth	45	44	43	42	41	31	32	33	34	35
Buccal Cusp	2.05	1.75	2	1.4	1.7	1.85	1.9	1.25	1.5	1.85
Palatal Cusp	1.85	1.15	–	–	–	–	–	–	1.8	2.0

On review of his medical history and on physical examination, the chest X-ray showed that the patient spine was slightly S-shaped, the S1 lamina was not closed with an irregular shape, and there was an uneven density between the metaphysis and the proximal femur on examination on November 13, 2019 (Fig. 1c1, c2). Later, the patient had undergone surgical treatment for chondroma of the legs (Fig. 1c3, c4).

In April 2020, compared to a height of 106.5 cm and weight of 18.3 kg at the age of 4 years and 10 months, the patient was 134 cm in height and weighed 42 kg; his weight had increased faster than his height which had increased slowly (Fig. 3A, Table 1). His fingers and toes were thick and short (Fig. 3B). However, this was not inherited, as his father, mother, grandfather and grandmother were 182 cm, 160 cm, 176 cm and 168 cm tall, respectively (Additional file 2: Fig. S2).

### Further treatment plans

The further treatment plans include orthodontic assisted traction of impacted teeth and implant restoration of missing teeth, with multidisciplinary joint-effort from different fields, such as pediatrics, surgery, and stomatology, for good functional and esthetic reconstruction of the patient.

## Discussion and conclusion

This above case presents a comprehensive course history of a patient with *FGFR1* gene mutation and OD syndrome which present with delayed teeth eruption. During our intervention and interaction with the patient, we have gained further insight into this rare case.

Reported literature reveal that mutations in the *FGFR1* gene, which controls the growth of bone and tooth formation and eruption, may be the main cause of OD syndrome [2–4], and as mentioned before, in terms of their role in tooth development, mutations at the site of *FGFR1* prevent the eruption of both deciduous and permanent teeth, which may be caused by the reversing and crookedness of teeth, the impact of hyperplastic gingiva, or even jaw cysts [2, 11]. The mutation also affects the number of tooth buds, resulting in the oligodontia recorded in the literature [7, 11]. In this case, the patient had enamel, dentin hypoplasia and decreased thickness, with a normal number of unerupted teeth. Despite the fact that many patients with OD have shown tooth eruption disorder, there is no treatment for it described in the literature, and the mechanism of OD remains unknown.

Patients can inherit OD through an autosomal dominant pattern; however, most of the cases of OD documented in the literature including this case usually have no family history, except for father-to-son transmission in two cases [3, 6]. The proband suffered from OD showed the same skeletal syndrome as his father and brother in one case [3], and the other, previously reported by Kelley et al. in 1983, suggests autosomal dominant inheritance of OD were found in the father and son who were both affected by skeletal dysplasia. As for our case, based on gene sequencing, the parents of this patient are both healthy non-carriers. Physically, his father, mother, grandfather and grandmother were 182 cm, 160 cm, 176 cm and 168 cm in height, respectively. This patient therefore, presented a case of spontaneous mutation and disease course.

Accordingly, the treatment of OD becomes difficult because cases are rarely seen and are variable. The complexity of OD involves many deformities, including the skeleton, teeth and even soft tissue. Treatment options include both medical and surgical approaches. In particular, it should be noted that the impaction of the teeth in the maxilla and mandible are consistent burdens for patients with OD, and awareness of this should be emphasized. There is no doubt that almost all of the impacted teeth cannot erupt by themselves, as reported in the literature, which is further confirmed by our 4-year follow-up case. In this case, although there were multiple attempts to assist the well-developed deciduous incisor by cutting the gums, they ultimately failed for all attempts were quickly-wrapped by gum, which may be induced by the mutations in the *FGFR1* gene for its ability to promote fiber proliferation. This also inspired us in our measures in preventing the gum from quickly wrapping to erupt the impacted teeth. Besides, it could be noted that the process of tooth eruption is under the precise coordination of dental follicles, tooth germ cells, osteoclasts, and osteoblasts with cascade molecular reactions; any interference of factors comprising congenital and acquired elements may bring about hypoplasia of teeth or failure in tooth eruption.

Tooth eruption disorders can be divided into mechanical failure of tooth eruption (MFE) and primary failure of tooth eruption (PFE), which dictate different treatment measures that should be considered [23]. Usually, only a single tooth or partial teeth are affected by mechanical factors in cases of MFE, which can be corrected by orthodontic traction. Patients with PFE are characterized by unilateral/bilateral open bite due to the disturbed eruption mechanism of non-ankylosed teeth, which show no response to orthodontic forces and familial aggregation, is also very common [23]. These disorders do not include the impacted teeth of OD, and their response to forces remains unclear. Limited studies have shown treatment options for the construction of prostheses and corrective surgery for craniofacial deformities that should be taken into consideration; unfortunately, there are wanting amount of literature showing their therapeutic effects and prognosis.

Because of the great pain, discomfort and other adverse effects caused by anodontia, as seen in our case, practitioners should make efforts to guide eruption of well-developed teeth depending on the individual clinical situation.

We here demonstrated a case of impacted teeth under the diagnosis of OD with detailed analysis of the patient’s dental and general conditions, which illustrates the importance of careful history taking and comprehensive knowledge as well as examinations in patients with delayed teeth eruption. In particular, it should be noted that the impact of the teeth in the maxilla and mandible are consistent burdens for patients with OD, which shall be noted for wider awareness.

## Supplementary Information

**Additional file 1: Fig. S1**. The facial and oral photos of his mother (A) and father (B) as well as their panorama films (C).

**Additional file 2: Fig. S2.** A genealogic tree of the family.

**Additional file 3: Fig. S3.** The complete denture of the patient.

## Data Availability

All data generated or analyzed during this study are included in this published article.

## Abbreviations

OD: Osteoglophonic dysplasia
*FGF*: Fibroblast growth factor
*FGFRs*: Fibroblast growth factor receptor
CCD: Cleidocranial Dysplasia
HED: Hypohidrotic Ectodermal Dysplasia
MFE: Mechanical failure of tooth eruption
PFE: Primary failure of tooth eruption
