# Supplementary figures and images for: Abnormal eruption of teeth in relation to FGFR1 heterozygote mutation: a rare case of osteoglophonic dysplasia with 4-year follow-up
Source: BMC Oral Health. 2022 Feb 11;22:36. doi: 10.1186/s12903-022-02069-6 (PMC8832749; doi:10.1186/s12903-022-02069-6)

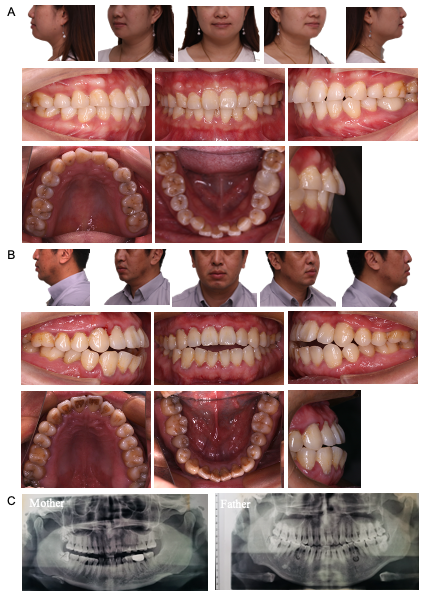

Supplement: Supplementary file 1 — Additional file 1: Fig. S1. The facial and oral photos of his mother (A) and father (B) as well as their panorama films (C). [file 12903_2022_2069_MOESM1_ESM.tif]

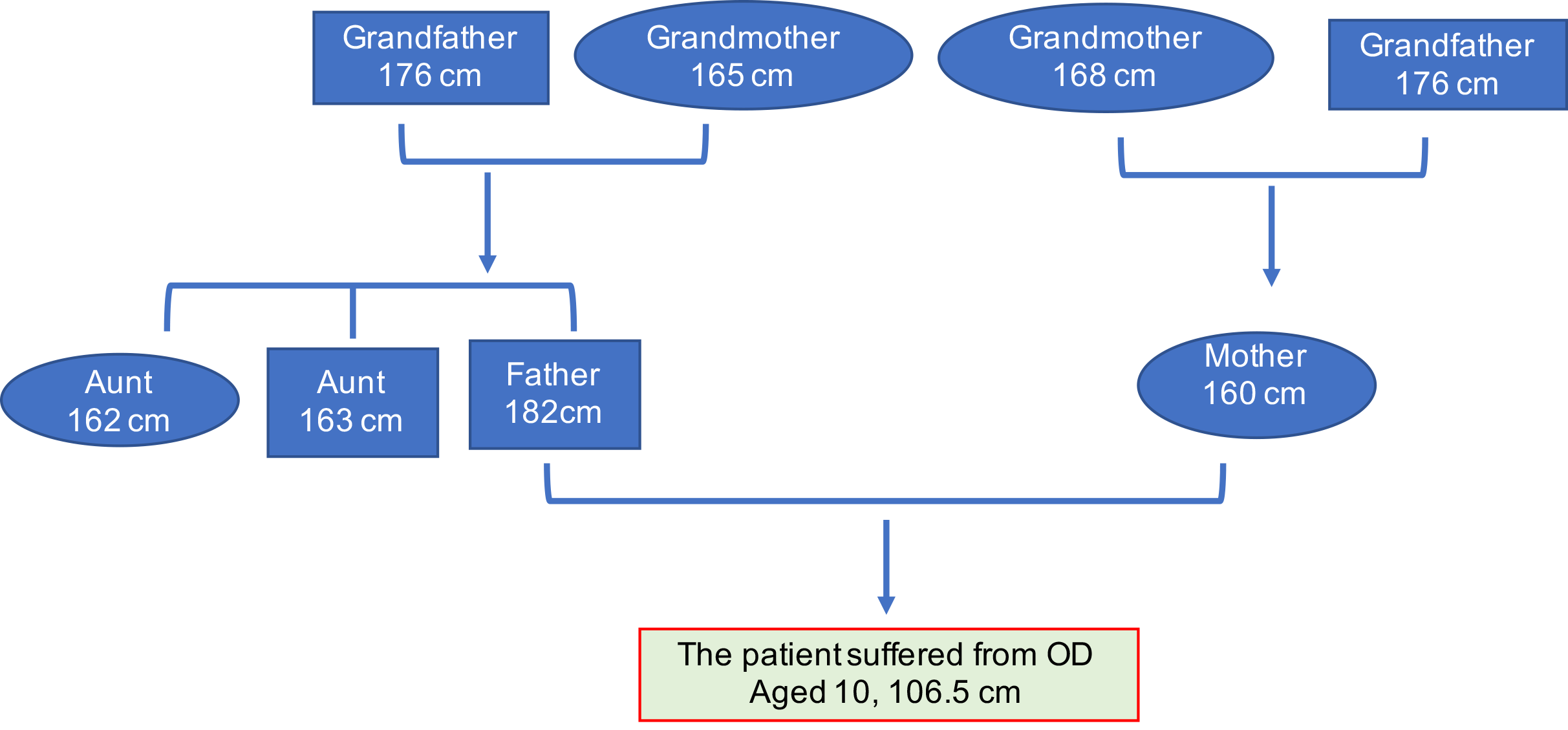

Supplement: Supplementary file 2 — Additional file 2: Fig. S2. A genealogic tree of the family. [file 12903_2022_2069_MOESM2_ESM.tif]

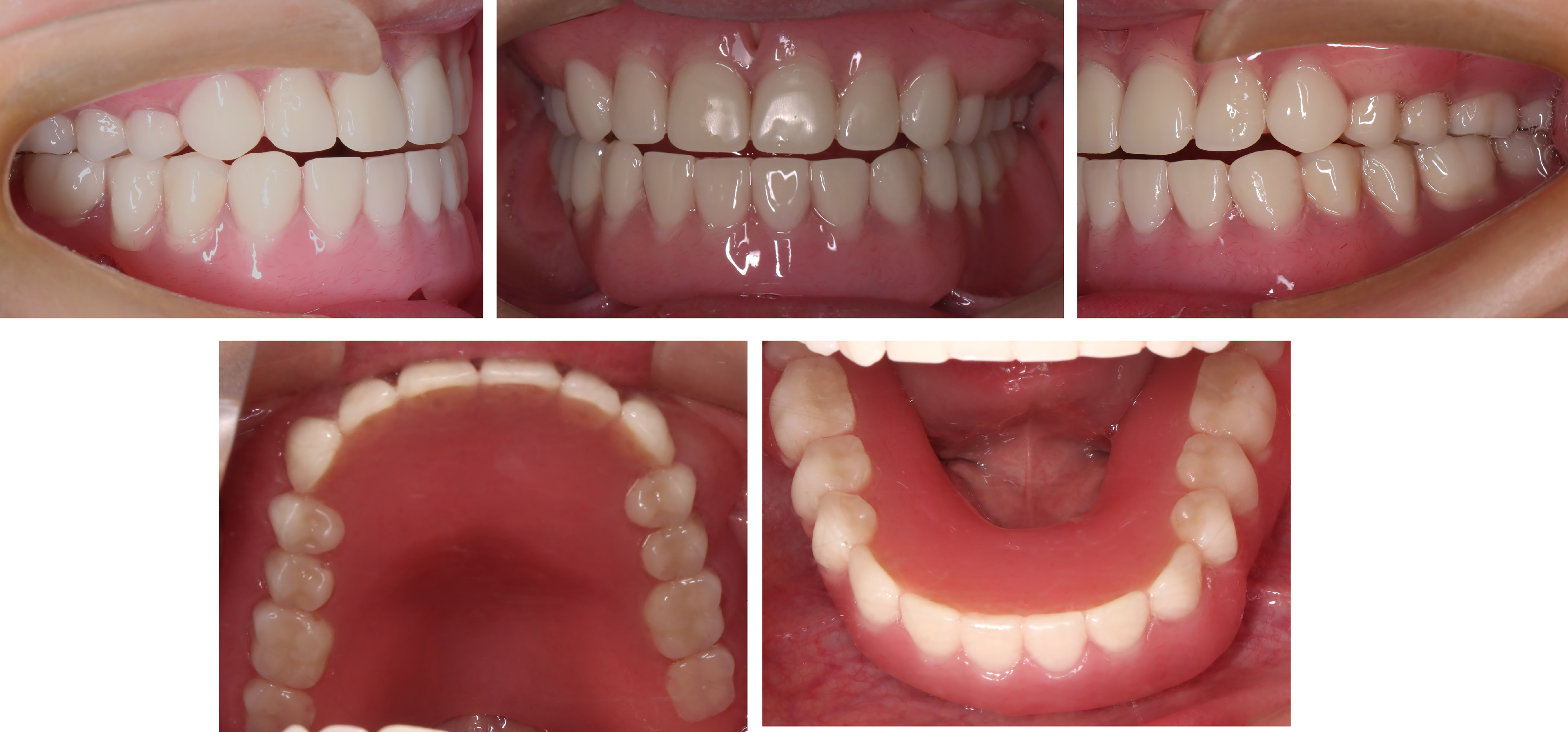

Supplement: Supplementary file 3 — Additional file 3: Fig. S3. The complete denture of the patient. [file 12903_2022_2069_MOESM3_ESM.tif]
